# Additively Manufactured Scaffolds for Bone Tissue Engineering and the Prediction of their Mechanical Behavior: A Review

**DOI:** 10.3390/ma10010050

**Published:** 2017-01-10

**Authors:** Xiang-Yu Zhang, Gang Fang, Jie Zhou

**Affiliations:** 1 Department of Mechanical Engineering, Tsinghua University, Beijing 10004, China; zhangxiangyu0012@163.com; 2 State Key Laboratory of Tribology, Beijing 100084, China; 3 Department of Biomechanical Engineering, Delft University of Technology, Mekelweg 2, 2628 CD Delft, The Netherlands

**Keywords:** additive manufacturing, scaffold, biomaterial, geometric design, mechanical property, finite element modeling

## Abstract

Additive manufacturing (AM), nowadays commonly known as 3D printing, is a revolutionary materials processing technology, particularly suitable for the production of low-volume parts with high shape complexities and often with multiple functions. As such, it holds great promise for the fabrication of patient-specific implants. In recent years, remarkable progress has been made in implementing AM in the bio-fabrication field. This paper presents an overview on the state-of-the-art AM technology for bone tissue engineering (BTE) scaffolds, with a particular focus on the AM scaffolds made of metallic biomaterials. It starts with a brief description of architecture design strategies to meet the biological and mechanical property requirements of scaffolds. Then, it summarizes the working principles, advantages and limitations of each of AM methods suitable for creating porous structures and manufacturing scaffolds from powdered materials. It elaborates on the finite-element (FE) analysis applied to predict the mechanical behavior of AM scaffolds, as well as the effect of the architectural design of porous structure on its mechanical properties. The review ends up with the authors’ view on the current challenges and further research directions.

## 1. Introduction

Bone tissue, or osseous tissue, is a major structural and supportive connective tissue of the body. Actually, it is a complex composite material that exists on at least five different hierarchical levels [1], namely whole bone level, architectural level, tissue level, lamellar level and ultrastructure level. At a microscopic structural level, bone can be roughly divided into two types: cancellous bone and cortical bone. Cancellous bone, i.e., the inner part of bone, has a spongy structure with varying porosities between 50% and 90% and consists of a large number of trabecula. Trabecula grows naturally along the stress direction, allowing the bone to withstand the maximum load with a minimum bone mass. Cortical bone, i.e., the dense outer layer of bone with a porosity of less than 10%, on the other hand, is highly compact and orthotropic due to the circular nature of the osteons that make up its structure.

Despite high mechanical strength, bone may be damaged and fracture may occur. Thanks to the high regenerative capacity of bone, particularly in younger people, the majority of fractured bones will heal by themselves without the need of major intervention. However, a large bone defect, for example, as a result of bone tumor resection, or severe nonunion fracture, needs an implanted template for orchestrated bone regeneration. Generally, bone remodeling goes through five stages: resting state, activation, resorption, reversal and formation [2]. Osteoblast and osteoclast are the two types of cells involved in the physiological processes of repairing broken bones. Bone naturally possesses the characteristic of mechanotransduction and trabecula grows in the direction of the principal stress. It is now widely acknowledged that loading magnitude and frequency have significant effects on bone remodeling. The main reason for osteopontin up-regulation is shear stress [3] and osteocytes play the role of mechanosensory cells that react to mechanical stimuli [4]. It is the distinctive and complex mechanotransductive growth mechanism of bone that poses a serious challenge to scaffolds for bone tissue engineering (BTE), with the intricate physiological environment of bone taken into consideration.

Currently, the gold standard treatment of a large bone defect is still the use of autografting, involving the harvest of donor bone from a non-load-bearing site in the patient. However, in recent years, engineered bone tissue has been increasingly viewed as a viable alternative to autograft or allograft, i.e., donated bone, due to unrestricted supply and no disease transmission. However, despite the promise that the BTE approach holds, it has not entered the large-scale clinical application phase, mainly because several major challenges have not yet been overcome. As the success of this approach depends on porous 3D scaffolds that are required to provide mechanical support and an appropriate environment for the regeneration of bone tissue, the design and fabrication of porous scaffolds with biocompatibility, desired architecture, mechanical properties and bioresorbability are some of the key challenges towards their successful implementation in BTE.

A BTE scaffold is actually a porous structure that acts as a template for bone tissue formation. Typically, the scaffold is seeded with cells and occasionally with growth factors and may be subjected to biophysical stimuli in the form of a bioreactor. The cell-seeded scaffold is either cultured in vitro to synthesize tissues and then implanted into the injured site, or implanted directly into the injured site to regenerate bone tissue in vivo by using the body’s own systems. To perform the desired mechanical and biological functions, it should exhibit excellent biocompatibility and the properties of extracellular matrix (ECM), such as mechanical properties, cellular activity and protein production through biochemical and mechanical interactions throughout the whole bone healing process [5] that is deemed dynamic and complex. The architecture of a scaffold in terms of porosity, pore size and pore interconnectivity is of critical importance, because it strongly affects the cellular activities and the mechanical properties that are needed for the scaffold to bear load, transfer load and match the host bone tissues both in Young’s modulus and compressive strength. In addition to the critical importance of scaffold’s geometry, in vitro and in vivo studies have demonstrated that the combination of additively manufactured polymeric and composite BTE scaffolds with autologous bone marrow-derived mesenchymal stem cells or mesenchymal progenitor cells or bone morphogenetic protein significantly promote the bone regeneration at a segmental bone defect [6,7,8].

A variety of synthetic materials may meet part of the requirements of BTE scaffolds. However, the failure to meet other requirements may disqualify them as suitable scaffold biomaterials. Inorganic bioceramics, for example, tricalcium phosphate (TCP), have desired bioactivity and biodegradability, but their brittle nature means that their fracture toughness cannot match that of bone and therefore these bioceramics are not suitable for load-bearing scaffold applications [9,10]. Synthetic polymers allow for easy scaffold fabrication to create regular porous structures with desired porosities and other geometric characteristics, for example, by means of fused deposition modeling [11,12], but most of polymeric scaffolds show rapid strength degradation in vivo and the degradation of biopolymers, such as polylactic acid (PLA) and polyglycolic acid (PGA), leads to the formation of a local acidic environment that has adverse tissue responses [13]. Metals have high compressive strengths and excellent fatigue resistance, but most of metals are not biodegradable and thus cannot create additional space, while being implanted, for the new bone to grow into and take over mechanical and biological functions. Biodegradable metals and alloys based on magnesium, iron and zinc are currently under development. The common concerns about metal ion release into the body fluids are being addressed. Obviously, the biomaterials that are currently available all have one or two deficiencies and therefore cannot fully meet the whole set of the requirements of BTE scaffolds.

In addition to the biomaterial challenge, there is an interconnected issue of scaffold fabrication. A BTE scaffold fabrication method, being specific to the chosen scaffold material, either metal, polymer, ceramic or composite, must be able to generate the desired architecture and ensure specified mechanical properties in a reproducible manner.

A number of traditional materials processing technologies have been adopted to fabricate metallic porous scaffolds in BTE studies, such as sintering of metal powder [14] or metal fiber [15], polymeric sponge replication [16,17], investment casting [18], and gas foaming [19]. Some of these technologies have been successfully implemented at a commercial level, e.g., tantalum orthopedic implants [20,21]. However, most of the technology development has been at the stage of demonstrating proof of concept in laboratory settings. For example, porosity-graded pure titanium compacts [22] and porous titanium structures possessing porosities ranging from 5.0% to 37.1% [23] were fabricated using powder sintering. Pores retained in the sintered compacts were interconnected and three-dimensional. The porosity and mechanical properties of the porous titanium structures were controlled by changing powder particle sizes and sintering condition. A novel technique, namely immersion of polymer sponge in TiNi slurry, was applied to fabricate TiNi scaffolds with relatively low Young’s modulus [24] and after sintering the scaffolds had porosities of 65%–72%. The compressive strength and Young’s modulus values of the scaffolds achieved were similar to those of cancellous bone. In most of the traditional processes, pores are generated by means of a foaming agent, or inner gas blowing, or partially melting of metal powder/fiber, which means that it is very difficult to control the porosity and the geometry, sizes and interconnectivity of pores inside the scaffold, which may lead to irregular inner structures and cause severe stress concentration and shortened fatigue life. Although polymeric sponge replication and investment casting show a better controllability over the internal porous structure, many negative issues are yet to be addressed, such as the toxicity of additives, control of the drying process, the difficult polymerization process and process complexities. Therefore, developing new fabrication methods for metallic scaffolds is badly needed.

Additive manufacturing (AM) is a new materials processing method based on a three dimensional (3D) CAD model to fabricate a part, or an integrated part, in an additive manner, mostly layer by layer, without the need of process plan as that involved in the conventional fabrication processes [25]. Since the 1980s, AM technologies have gained great attention, as they have shown obvious advantages over the traditional subtractive fabrication technologies. Due to the ability to fabricate extremely complex parts without any tools or molds, this game-changing manufacturing technology has been used in the research community to create patient-specific implants for bone substitution. AM has proven itself to be a viable process to fabricate metallic BTE scaffolds, from a variety of metal powders or alloy powders, such as Ti [26], Ti-6Al-4V [27,28], Fe-30Mn [29] and Ta [30]. Most of the AM processes applied to the fabrication of BTE scaffolds are powder-bed-based or powder-fed-based ones. For example, Murr et al. [31] fabricated patient-specific biomedical implants by electron beam melting (EBM). Wauthle et al. [32] made use of selective laser melting (SLM) to make load-bearing scaffolds. The scaffolds so fabricated possessed great biocompatibility, biological and mechanical functions, and even biodegradability when a biodegradable metal such as magnesium or a biodegradable alloy was used [33]. A large portion of the research on AM for BTE scaffolds has been focused on their mechanical properties, such as Young’s modulus, yield strength, tensile/compressive strength and fatigue behavior. In addition, a number of studies concerning in vitro and in vivo assessments of AM scaffolds have been conducted [30,34,35].

In order to achieve the desired mechanical properties of scaffolds and the desired architecture for nutrition supply as well as for drug delivery, different regular unit cells have been proposed. Finite element (FE) models have been developed to predict the mechanical properties of a chosen porous structure, for example, its fatigue behavior. Relevant experiments have been conducted to validate the FE simulation results [36,37,38,39].

In this review, we first introduce the basic AM technologies for BTE scaffolds, with a focus on the mechanical properties of metallic scaffolds fabricated by means of AM technologies and FE studies to correlate the mechanical properties with architectural design.

## 2. Requirements of BTE Scaffolds

Currently, BTE is still at the early stage of research in the laboratory and animal models are often used; BTE practices have not proceeded to clinical practice, due to the complex nature of BTE. Scaffold design and fabrication are the two integrated elements of BTE. An ideal BTE scaffold should enable osteogenitor cells to attach, proliferate and differentiate into functional bone tissue, i.e., to serve as a growth matrix for bone cells. To be more specific, BTE scaffolds are expected to have the following five characteristics:

- Good biocompatibility;
- Appropriate pore sizes and porosity that are suitable for bone cell infiltration and growth;
- Comparable mechanical properties with adjacent bone tissue;
- Osteoconductivity and osteoinductivity;
- Biodegradability. When the bone defect is healed, there should be no traces of the original prosthesis. The degradation products should have no side effects on the human body.

Young’s modulus is considered to be one of the most significant characteristics in the biomechanical research on BTE scaffolds, on top of sufficient compressive strength to bear osteogenic loads during healing. Furthermore, with the application of the AM technology, the geometrical structure of the scaffold can be precisely controlled and targeted mechanical and biological properties can be achieved through functionally graded architectures. Scaffolds with pore sizes ranging from 300 to 400 μm were found to cause a remarkable improvement in bone tissue recovering [40]. The promoting effect on bone regeneration increased with increasing sizes of pores that were in the near-bone area of the scaffold. In order to enhance the controllability of the inner architecture of scaffolds and further improve their mechanical properties, nutrient transportation and drug loading ability, spatial-periotic structures composed of hollow polyhedron unit cells have been proposed to be a favorable design scheme [37,38,39,41]. Arabnejad et al. [30] proposed a method to determine pore size, strut diameter and porosity based on the requirements of the overall performance of the scaffold and the limitations of a particular AM technology.

**Figure 1:**
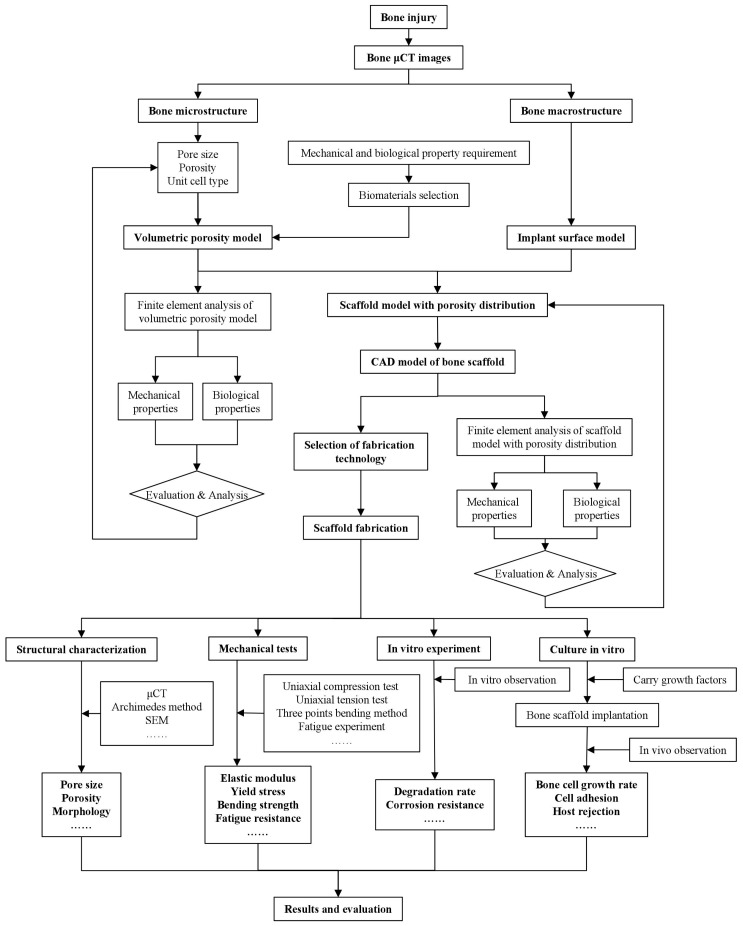
Procedure of design, fabrication and evaluation of BTE scaffolds. μCT, micro-computed tomography.

An overview of the whole BTE procedure including evaluation through in vitro cell culture is shown in Figure 1. First, a scaffold structure with a set of geometrical features including internal pore characteristics and a personalized external shape is designed, according to the anatomic structure of the bone at the defect site. Then, an appropriate biomaterial and a fabrication method are selected to produce the scaffold. After post-processing to modify the surface for enhanced cell attachment and biocompatibility, the scaffold is cultured in vitro with growth factors and bone marrow cells for a sufficient length of time. Once implanted, the scaffold biodegrades gradually and new tissue grows simultaneously. Post-operative monitoring takes place by means of imaging techniques, such as computed tomography (CT). For research purposes, the implanted scaffold may be retrieved for further evaluation and analysis.

From a biological point of view, a BTE scaffold provides appropriate mechanical stimuli for osteoblasts and osteoclasts and activates bone growth mechanisms. The selection of the fabrication method is based on the need to fulfil the biological and mechanical property requirements of the scaffold; the creation of a porous structure with desired porosity, pore sizes and pore interconnectivity as well as mechanical properties is often followed by surface bio-modification with minimum adverse side effects on the mechanical properties.

## 3. Additive Manufacturing of Metallic BTE Scaffolds

As mentioned above, AM is a technology to construct 3D components based on a layer-upon-layer methodology, as opposed to the traditional subtractive manufacturing technologies. With this materials processing technology, complex digital 3D designs can be turned into functional physical objects efficiently and precisely. Anoft-quoted example of the application of the AM technology is the freeform fabrication of jet engine fuel nozzles by General Electric [42]. Typically, a STL (STereoLithography) file can be created in one of the following three ways:

- Importing a CAD (Computer-Aided Design) file to an AM system and slicing the original model into layers;
- Using reverse engineering or CAD method to obtain design model data in the STL format and then slicing the model into layers;
- Analyzing and reconstructing a target structure based on medical CT or MRI (Magnetic Resonance Imaging) images.

With the rapid development of the AM technology in recent years, AM has been increasingly used in the research on BTE. By making use of AM technology, precise control of the architecture and structure integrity of scaffolds become possible by adjusting the processing parameters. It is, however, important to note that the correlations between individual geometric parameters and the mechanical behavior of scaffolds are highly complex and difficult to establish. For most of metal AM processes, residual stresses are present in the as-printed scaffolds and non-equilibrium phases may remain in the as-printed microstructure. Post-processing for stress relieving and phase transformation is often needed. In comparison with metal AM, post-processing is a much neglected area of research.

### 3.1. Brief History of AM Technologies

The concept of manufacturing parts layer by layer was proposed at the end of the 19th century. The technology originated in the United States was first used in photo sculpture and topographical maps. In the late 1980s, Mr. Chuck Hull developed an AM process that could translate numerical data into 3D objects by making use of the stereolithographic technology (SLA). Shortly afterwards, Mr. Scott Crump founded 3D Systems. The world’s first fused deposition modelling (FDM) machine was invented by Stratasys in 1991 [43]. Dr. Carl D. Deckard and his colleagues at the University of Texas developed the selective laser sintering (SLS) technology [44], which makes use of a moving laser beam to trace and selectively sinter thermoplastic plastic, metal and ceramic powder into successive cross sections of a 3D object.

The past 15 years have witnessed the transformation of additive layer manufacturing technologies from rapid prototyping mostly for product development to AM for end-use part production. Laser-based or electron beam-based AM technologies have brought about a game-changing revolution in industrial manufacturing, especially in the biomedical application field. Nowadays, the ranges of products and materials are rapidly growing and the complexity and accuracy of AM parts are being noticeably improved.

### 3.2. Category of AM Methods

In general, AM technologies can be classified into several categories, according to the raw material feed system (e.g., powder-bed, powder-fed, or wire-fed) and the energy source (e.g., laser, electron beam, or plasma arc). American Society for Testing and Materials (ASTM) International Committee classified major AM technologies or 3D printing technologies into seven groups. In this review, we summarize the major AM technologies relevant to the fabrication of BTE scaffolds, together with their features in Table 1. In most of the research on metallic BTE scaffolds, selective laser melting and electron beam melting have been selected to be the preferred scaffolds fabrication methods because of their good controllability and high precision, while other direct energy deposition methods such as direct metal deposition (DMD) and three-dimensional printing (3DP) generally possess the characteristics of lower processing accuracy (380–16000 μm) and larger layer thickness (250–3000 μm) [45], which would restrict their applications to large part fabrication and reparation. Table 2 qualitatively compares the characteristics of these AM technologies.

### 3.3. Metal or Alloy Powder Precursor

In the powder-bed-fusion technologies such as SLM and EBM, the raw materials are often in the form of fabricated powder particles. It is the first step in the manufacturing of metallic scaffolds. Powder particles suitable for AM should possess a proper particle size distribution and morphology. There are quite a large number of other important characteristics that need to be taken into consideration in selecting metal powder and its fabrication method, including chemical composition, flowability, apparent density, thermal properties, electric properties, and laser/electron beam energy absorption capacity. Among these characteristics, chemical composition and powder particle size distribution are by far the most crucial ones. The chemical composition of a metal powder is often analyzed through chemical analysis or spectral analysis. The particle sizes of currently used powders range from 15 to 150 μm. Energy source such as laser beam is mostly adopted in fine powder AM processing, while plasma beam is more preferable when powder particle size is larger.

New powder fabrication methods, such as powder manipulation technology (PMT) developed by the Commonwealth Scientific and Industrial Research Organization (CSRIO) of Australia, offer the possibility to manipulate the size and shape of low-cost powder for AM, e.g., through high shear milling of sponge titanium into a low-cost titanium powder precursor [55]. A novel powder precursor with more than 50 wt % of particles in the particle size range of 45 to 160 μm and 30 wt % of particles with sizes less than 45 μm was produced. There are a number of issues related to powder feedstock, specific for AM processes, such as powder reuse and powder removal. Tang et al. [56] investigated the effect of powder reuse time on the characteristics of Ti-6Al-4V powder and found that powder particle morphology, chemical composition, particle size distribution and flowability would significantly change after 16 or even more reuse times.

For medical applications, titanium alloy scaffolds are often made by using electron beam manufacturing, thus inevitably leaving powder particles trapped within porous structures. Hasib et al. [57] evaluated a chemical etching process for trapped powder removal from Ti-6Al-4V cellular structures with pore sizes of <600 μm and found difficulties of removing trapped powder without affecting the integrity of the porous structure. With the laser-based AM methods, however, powder entrapment is not an issue, because of a relatively low working temperature.

### 3.4. AM Standards and Norms for Medical Applications

Along with the maturing of the AM technology and growing industrial interest, standardization to set technical or quality requirements that various AM products, AM processes, services or methods may comply with has been increasingly recognized as an essential component of AM development. Some harmonized standards for AM design, materials, processes, terminology and test methods have already been established by the American Society for Testing and Materials (ASTM) and International Organization for Standardization (ISO)—the two globally recognized leaders in the field of international standards. For example, the standards for the determination of metallic powder properties (powder sampling, sizes and size distribution, morphology, flow behavior, thermal characteristics and density), the standards of AM with powder bed fusion for Ti-6Al-4V (ASTM F2924-14, ASTM F3001-14) and stainless steel alloy (ASTM F3184-16) and the standards of mechanical testing of porous and cellular metals (ISO 13314:2011) have been developed.

In addition, a number of tissue engineering (TE) standards, such as ASTM F2211-13, ASTM F2312-11 and ASTM F2150-13, have been issued, which systematically define the practices related to biomaterials manufacturing, application and evaluation. However, so far, the AM standards and TE standards have been established independently and the standards specific on medical devices and implants fabricated using AM technologies are still missing [58]. Therefore, developing a sound and complete system of standards and norms for additive biomanufacturing is in urgent need.

## 4. Architectural Design of BTE Scaffolds

The strength and stiffness of metallic materials are much higher than those of human bone. Thus, the dense metallic scaffold will bear most of the loading after implantation. Subsequently, the stress level of adjacent bone tissue is dramatically decreased and the problems of bone resorption and implant loosening are induced. The design strategy of porous structures can effectively reduce the strength and stiffness of scaffolds and porous structures also provide sufficient space for new bone tissue ingrowth. At present, the mainstream way of non-stochastic cellular structure design is arranging structural units, such as polyhedral units or point lattice periodically to get a porous architecture. The structural units can be designed through CAD [30], image-based designing [59], implicit surface modeling [27,60] and topology optimization [61,62]. The geometrical shape of structural units reported in the literature can roughly be classified as truss, polyhedron and triply periodic minimal surface.

Ashby put forward cubic unit models for open-cell foam and closed-cell foam. A relationship between the porosity and overall mechanical properties was derived [63]. The results predicted by the models showed a good agreement with experimental data. This study gave a useful insight into human bone structure simplification and scaffold design. Cheah et al. [64,65] established a library containing eleven types of unit cells based on the considerations on the manufacturability related to specific AM technologies and on spatial geometry properties; each type of polyhedrons was repeated regularly in 3D space only by joining vertices or edges and connected at faces. Scaffolds consist of diamond lattice [66], cubic lattice [67], truncated octahedron [68], rhombic dodecahedron [69] and rhombicuboctahedron [70] were studied and the analytical solutions of the Young’s moduli and Poisson’s ratios of the scaffolds were expressed in terms of geometric parameters such as porosity, strut diameter and pore size. In order to further improve the accuracy of the analytical solutions, Ahmadi et al. [66] adopted the Euler beam and Timoshenko beam theories in the cases of the deviations of Young’s modulus, yield stress and Poisson’s ratio from experimental results and their results showed that the Timoshenko theory was preferable when apparent density was high.

It is noteworthy that the recovery after scaffold implantation is a two-stage process. At the initial stage of recovery, the type of material plays the most dominant role. At the second stage, however, pore size and shape become the crucial factors. No uniform conclusion about an optimum pore size has been reached. Pore sizes in a range of 200 to 500 μm were systematically studied [34,71,72]. Acceptable results were obtained with pore sizes that were smaller than 200 μm [73,74], larger than 500 μm [28,75,76] and even up to 2200 μm [77].

In addition to pore size and porosity, surface curvature has a significant impact on bone tissue regeneration. Previous studies showed that a concave surface was more beneficial for osteocyte attachment and proliferation than a flat or convex surface and a concave surface helps promote the migration of cells and improve the tissue morphology, including the expansion of the cell proliferation area [78]. Jinnai et al. [79] confirmed that the mean curvature of human trabecula was close to zero. Inspired by the natural trabecular structure and the zero-curvature feature of minimal surface, a more advanced structure unit named triply periodic minimal surface (TPMS) was developed. Yan et al. [27] fabricated Ti-6Al-4V alloy TPMS scaffolds by SLM; the scaffolds possessed pore sizes (480–1600 μm) and porosities (80%–95%) similar to those of trabecular bone and optimum mechanical properties could be obtained by tuning the SLM process parameters. According toGiannitelli’s summary about the future trend for BTE [80], implicit surface modeling is gaining more attention, the pore size and shape can be altered, and even graded porosity can be realized by modifying the implicit surface equations. Melchels et al. [81] compared the mechanical properties of gyroid TPMS scaffolds with those of stochastic scaffolds made by the particle-leaching method; their results showed that TPMS scaffolds could better promote the infiltration of cell suspension and tissue growth with the same porosity. Moreover, the permeability of TPMS scaffolds was ten times larger than that of the scaffolds made by the particle-leaching method.

Kapfer et al. [82] discussed a scaffold architecture with a sheet-like morphology based on minimal surfaces; these sheets were porous solids obtained by the inflation of cubic minimal surfaces to the sheets of a finite thickness, as opposed to the conventional network solids where the minimal surface formed the solid/void interface. Sheets possessed better mechanical properties and larger surface area.

In the treatments of segment defects of long bone, in order to mimic the original bone shape, morphology and overall physiology fully, scaffolds should possess the characteristics of gradient porosity and function, and even changing unit cell types. Functionally graded scaffolds (FGSs) are porous biomaterials, in which porosity changes in space with a specific gradient. Huang et al. [83] designed an anisotropic scaffold through adjusting the ratio of the semi-major axis to the semi-minor axis of prolate spheroidal pores. The hybridization CAD designs of TPMS FGSs were programmed by making use of Mathematica 9.0 [84,85] and the sigmoid function and Gaussian radial basis function were applied to simple transition boundary cases and general cases, respectively. In order to facilitate the subsequent AM, pores with gradient sizes, types and orientations and different porosities can be integrated to create a single architecture and exported as an STL-file.

Considering the fact that there are still no widely accepted descriptors of periodic trusses, Zok et al. [86] laid out a system for the classification of truss structure types. In their study, the concepts of crystallography and geometry were adopted to describe nodal locations and connectivity of struts.

**Figure 2:**
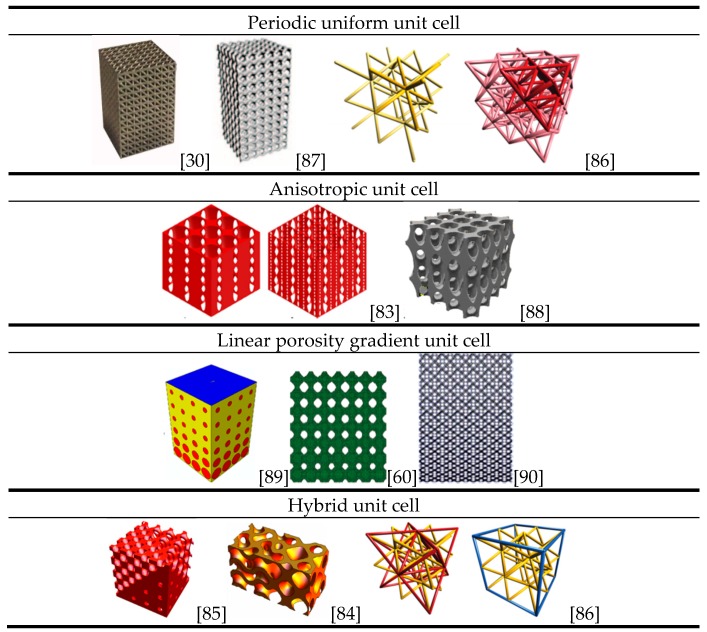
Scaffold designs based on different types of unit cells.

Figure 2 shows a variety of porous structures based on different types of unit cells [30,60,83,86,87,88,89,90]. Note that a general rule of unit cell selection and scaffold design is still missing and a universal characterization method for porous structure is in urgent need.

## 5. Computational and Experimental Studies on BTE Scaffolds

### 5.1. FE Modelling to Predict the Mechanical Behavior of Scaffolds

To understand the mechanical responses of scaffolds during their service life, various FE models have been adapted and further developed. In the earlier studies on the mechanical response of bone, FE models demonstrated their capabilities in tackling bone loading problems [91,92,93]. The work of Smith et al. [94] opened up a way to predict the mechanical properties of lattice structures by simulating a small number of unit cells of non-stochastic cellular materials.

Recently, two approaches to FE modeling have emerged, i.e., based on the micro-computed tomography technology (μCT) and the optimized model with manufacturing irregularities incorporated. It has been realized that the final parts manufactured by the AM technology often differ from the corresponding CAD models. Therefore, the first approach is to use the μCT technology to remodel the porous structure and then predict the mechanical behavior of an additively manufactured scaffold. Barui et al. [95], for example, adopted the μCT technology to determine the porosity and interconnectivity of Ti-6Al-4V scaffolds fabricated by using inkjet-based 3D powder printing (3DP). A FE model was established, based on the results of μCT analysis, and the compression properties predicted by FE simulation were found to corroborate reasonably well with experiment measurements. The research not only provided an insight into the global deformation behavior of the scaffolds but also depicted the local stress environment that the scaffolds were supposedly subjected to.

Another way to predict the mechanical properties of a lattice structure by using the FE method is using the original CAD model with or without considering the irregularities caused by the manufacturing process, including the structural variations of the architecture implemented. FE models of titanium alloy scaffolds considering manufacturing and material instability were developed by Campoli et al. [37]. Irregularities such as diameter variations in the cross-section area of the struts as well as the defects of the material were incorporated into the FE models, assuming a Gaussian distribution. Although the FE models showed a great accuracy in predicting the mechanical properties of porous materials, creating and using the FE models is in general more difficult since one needs to create a new FE model for each new material. Moreover, structural irregularities caused by AM processes must be implemented in the FE models because they may significantly influence the mechanical properties of porous scaffolds. Inspired by Campoli’s work, Zargarian et al. [96] constructed FE models of porous scaffolds based on three types of unit cells, namely rhombic dodecahedron, diamond and truncated cuboctahedron. Their fatigue failure behavior was investigated and the results indicated a failure plane at an angle of 45° to the loading direction. This work further illustrated the validity of this modeling approach. However, because AM process parameters have an intricate relationship with manufacturing defects and irregularities, a large number of experiments are still needed to determine more appropriate process parameters.

To account for the strut diameter differences between the design and AM products, as a kind of manufacturing irregularities, and to improve computing efficiency, Suard et al. [97] proposed a concept of equivalent diameter, based on the statistical analysis of the lattice structures fabricated by using EBM. The elastic response of a strut was represented by an equivalent cylinder. In their research, the equivalent diameter was significantly smaller than the nominal diameter, considering the fact that manufacturing defects and irregularities limited the load transfer ability of the cellular structure. Although the FE modeling results were specific to the particular condition of this study, the methodology used was general and could be applied to various AM processes.

**Figure 3:**
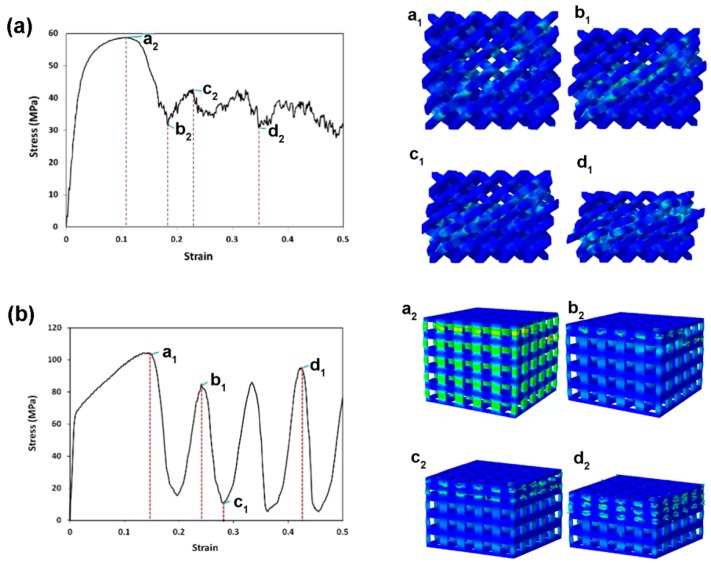
Failure mechanisms of (**a**) the diamond lattice structure at a 22% volume fraction; continuous shearing band of 45°, owing to crushing diagonal layers, is observed. Shearing of layers is accompanied by the bending failure of tying struts perpendicular to the diagonal plates; and (**b**) the cubic lattice structure; layer-by-layer deformation mechanism is confirmed by stretch-dominated deformation in scaling law analysis [98].

To understand the failure mechanisms of different lattice structures, as experimentally observed, Kadkhodapour et al. [98] implemented John-Cook plasticity and damage model in the cubic and diamond unit cell FE models based on the CAD design to simulate the failure behavior of Ti-6Al-4V scaffolds under compression. Their results obtained from FE simulations are shown in Figure 3. Failure was accompanied by the shear bands of 45° in the bending-dominated structures, i.e., the structures made from diamond unit cells, while layer-by-layer failure was seen for the stretch-dominated structures, i.e., the structures made from cubic unit cells. In addition, when the struts were designed to be placed parallel to the loading direction, buckling was excepted, resulting in the structure to experience the stretch-dominated deformation behavior, while the more inclination of micro-struts the more shearing failure was observed in the whole of the structure. Furthermore, when bending was dominated in the deformation of scaffolds, lower specific mechanical properties were expected, as compared with the stretch-dominated structures. Comparison of computational stress-strain curves with the experimental ones showed a good ability of the Johnson Cook damage model to predict the stress at the first peak as well as the plateau stress with a relative error less than 18%. Identical deformation mechanisms were also predicted by the models of TPMS-based scaffolds [60] and further validated by compression tests of the AM scaffolds, using FullCure850 and FullCure750 photopolymer resins as the printing and support material, respectively. It is worth noting that the scaffolds in this study had a linear gradient porosity and the bending tests showed brittle fracture at a strain of 0.08.

**Figure 4:**
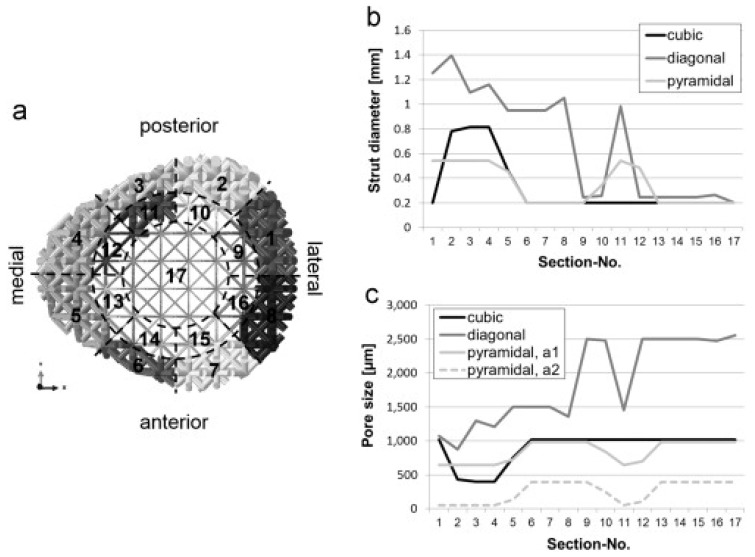
Distribution of the strut diameter of the 17 sections for the biomechanically optimized scaffolds with diagonal design (**a**); results in terms of strut diameter (**b**); and pore size (**c**) of each section for all the three investigated scaffold designs [100].

In an effort to optimize the lattice structure design against specific loading conditions, Wieding et al. [99] performed a numerical study on the scaffolds for large segmental defects and revealed that decreasing the amount of the inner core material had less influence than increasing the porosity when the scaffolds were loaded under biomechanical conditions. Wieding et al. [100] later on investigated numerically different CAD scaffolds composed of cubic, diagonal and pyramidal unit cells, using a numerical optimization approach. In their study, a large-size bone scaffold was designed and placed in a 30 mm segmental femoral defect site under a biomechanical loading condition. The strut diameter for the 17 sections of each scaffold was optimized independently in order to match the biomechanical stability of intact bone, as shown in Figure 4. This study provided a good example of optimized scaffolds for bone regeneration by considering both mechanical and biological aspects and using the numerical optimization approach.

**Figure 5:**
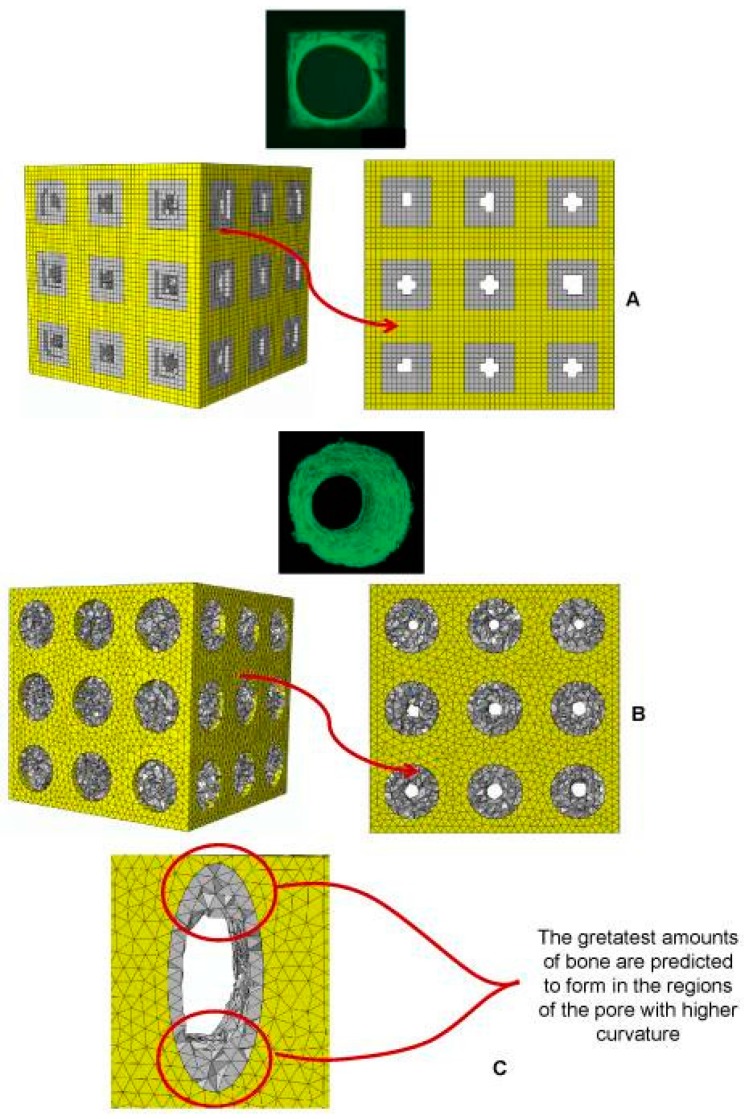
Patterns of bony tissue (3D view and frontal view) predicted by the optimization algorithm in the case of (**A**) square pores, under a pressure of 1 MPa and with a scaffold Young’s modulus of 1000 MPa; and (**B**) circular pores, under a pressure of 1 MPa and with a scaffold Young’s modulus of 1000 MPa; (**C**) a detailed view of the pattern of bony tissue predicted to form in an elliptic pore. The gray elements represent the volume within the scaffold where bone formation is predicted to occur [101].

The above cited numerical studies on uniform lattice structures clearly show the role that FE modeling can play in developing lattice design methods. The same strategy can be applied to develop the design methods for functionally graded lattice structures with changing porosity in space to better fulfill the mechanical and biological requirements for the regeneration of bone tissue, although this is computationally more demanding. Boccaccio et al. [89,101], for example, developed an algorithm combining the FE models of functionally gradient scaffolds, numerical optimization methods and a computational mechano-regulation model. Both shear strain and interstitial fluid flow were taken into consideration in the calculation of biological stimuli. The simulation results revealed that rectangular and elliptic pores could facilitate a larger amount of tissue growth than circular pores, and the fastest-growing bone tissue was found at the location where the curvature was the largest (Figure 5). These studies proved to be an efficient way for scaffold architecture optimization, when biological loading condition was considered.

The above mentioned studies all show that FE modeling is indeed an efficient tool for the research on the mechanical properties of BTE scaffolds affected by scaffold design. It is also clear that there is a great potential for FE modeling to predict the mechanical behavior of porous structures with a huge number of unit cells by modelling the constitutive unit cells to prevent the restrictions currently encountered in solving large models, provided that appropriate boundary conditions are applied. So far, numerical optimization of scaffolds considering biological loading has been highly time consuming and computational cost rises sharply when the model becomes complex. A multi-scale modeling strategy may be adopted to reduce the computation time and costs. To this end, during FE simulation, a scaffold is modeled as a fully dense material possessing material properties equivalent to those of a porous scaffold [102]. In the future, great efforts are needed in the following three interesting areas:

- Conducting FE simulations of scaffolds, considering biological loading and the flow of body fluid, as well as the reduction of artificial material.
- Improving the calculation efficiency and optimization methods of complex scaffold models for large segmental defects, for example, functionally gradient scaffolds.
- Developing FE models that can accurately simulate the AM processes involving powder melting and solidification during scaffold fabrication, in addition to predicting the mechanical properties of the resultant scaffolds accurately.

### 5.2. Metallic Scaffold ArchitecturalOptimization Based on Mechanical Property Analysis

With the recent development of AM technologies and proven biological functions of BTE scaffolds, more and more researchers have come to the realization that only open unit cell structures with controllable architecture and interconnected pores can allow the best performance in cell attachment, proliferation and differentiation and that such dedicated structures can only be realized by applying AM technologies. Compared with stochastic porous structures, regular porous structures have distinct advantages in mechanical property homogenization and osteoconductivity. Unit cell type is another key factor for the mechanical and biological properties of scaffolds; unit cell configurations such as cubic, diamond, truncated cube, honeycomb, etc. have been taken as typical examples in recent studies, although the underlying reasons for choosing these unit cell configurations are not specified. Among those studies, titanium and its alloys have been the most widely investigated materials for bone substitution, considering their excellent biocompatibility, corrosion resistance and good manufacturability for AM. Most of the studies have been focused on the mechanical performance of titanium or titanium alloy scaffolds [103,104,105,106], although the mechanical properties and AM technologies for scaffolds made of other alloys, such as stainless steel [103,107], Mg [108,109,110], Cu [107] and Ni [107] have also been investigated.

In the design of unit cell-based regular porous structures, Young’s modulus is taken as a key mechanical performance index for bone scaffolds. An ideal scaffold should have a stiffness value similar to the human bone. An increase or a decrease of bone mass strongly depends on the stress-strain state of the bone matrix [111]. After being implanted in the human body, the scaffold will not only bear the load caused by muscle action and gravity, but also facilitate and guide bone generation. During its service life, all the strain that the scaffold experiences should be limited to the elastic deformation region. Stress shielding can only be eliminated with an appropriate Young’s modulus and structure design. Although pore size, strut diameter and porosity can be tuned in the AM process, there exist some inherent limitations in doing so, because of manufacturing inaccuracy, metal powder inequality and post-processing. In addition, any change of one geometric parameter will inevitably cause changes in other geometric features of metallic scaffolds [112]. In many studies, scaffolds with different porosities were fabricated by changing pore size or strut diameter.

To verify the design idea to achieve a targeted stiffness value for a particular porous structure, uniaxial compression tests are widely used to determine Young’s modulus. The stress-strain curve is usually divided into three stages. The first stage is the elastic deformation stage. The second stage contains a stress plateau caused by elastic buckling and yielding. The third stage is also called the strengthening stage where the specimen is severely deformed and the inner architecture is crushed and struts become contacted with each other, leading to a sharp rise in stress. The compression test of a foamed aluminum structure indicated that the stress-strain curve was not strictly straight at the elastic stage; the specimen did not recover to its initial shape completely after unloading [113], which means that plastic deformation also occurred at this stage. The International Organization for Standardization [114] defines that the gradient of the elastic straight line is determined by the elastic loading and unloading between the stress of σ_70_ and the stress of σ_20_, and σ_70_ and σ_20_ are referred to as the plateau stresses at strains of 70% and 20%, respectively. By means of SLM or EBM, many researchers fabricated porous scaffolds composed of lattice truss or polyhedron. With appropriate pore sizes inside the scaffolds, Young’s modulus and porosity values similar to those of human trabecular bone were obtained. The stress-strain curve showed that the type of unit cell had a non-negligible effect on the mechanical properties of the scaffold. Generally, with an increase in apparent density, the stress-strain curve rises and fluctuations decrease.

**Figure 6:**
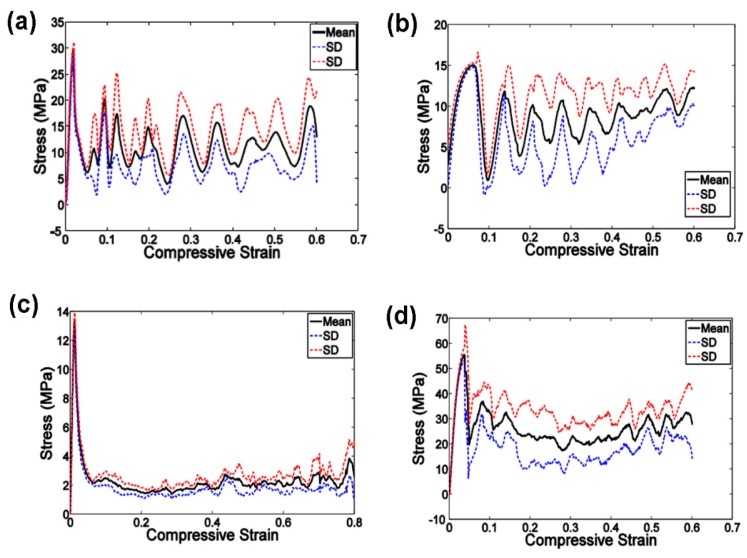
Compressive stress-strain curves of the specimens based on the cube unit cell and with porosity of (**a**) 88%; (**b**)78%; (**c**) 74%; (**d**) 66% [39].

To establish the relationship between the lattice structure type, porosity and mechanical properties, Ahmadi et al. [39] fabricated six types of Ti-6Al-4V space-filling unit cells with increasing relative density by means of SLM. Cylindrical specimens with a length of 15 mm and a diameter of 10 mm and a unit cell size of 1.5 mm were subjected to uniaxial compression testing. The results showed that the mechanical behavior, mechanical properties and failure mechanisms of these porous structures were strongly dependent on the type and dimensions of the unit cells investigated. Compressive properties of these structures increased with increasing relative density (RD). The stress-strain curves appeared to be distinctly different from those of the solid structure. Typically, the stress-strain curve started with an elastic deformation stage, followed by a stress plateau region and the subsequent fluctuations of the stress-strain curve. At the final stage of compression testing, the curve was often accompanied by the stiffening of the porous structure. The amplitudes of stress fluctuations generally decreased along with increasing relative density of the porous structure. All these mechanical characteristics can be observed in Figure 6.

**Figure 7:**
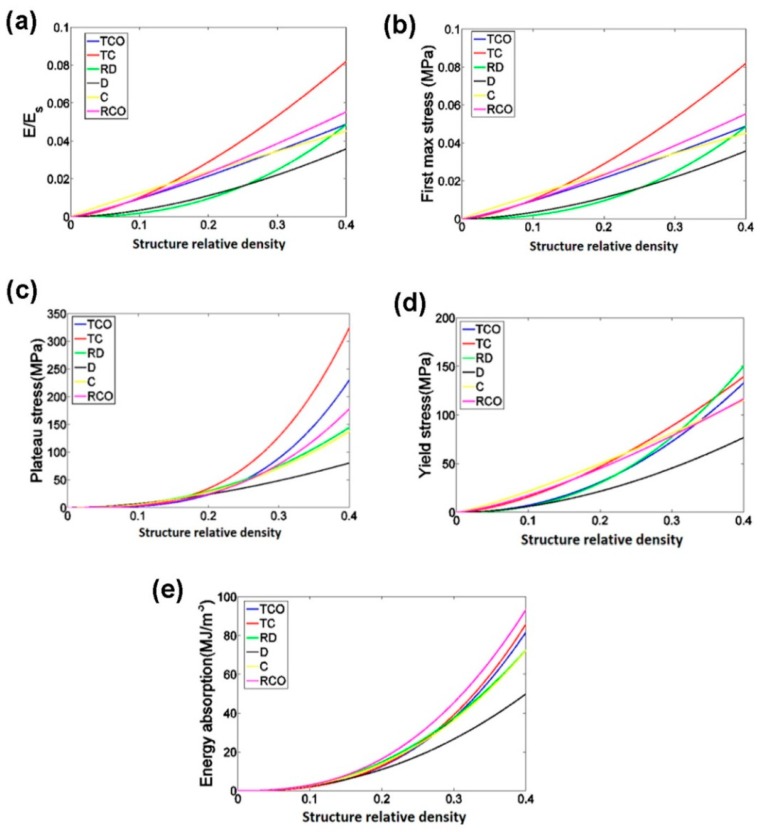
Comparison between the mechanical properties of different types of porous structures based on the six different unit cells: (**a**) elastic gradient; (**b**) first maximum stress; (**c**) plateau stress; (**d**) yield stress; (**e**) energy absorption. In these figures, the exponents of the power law fitted to the experimental data points, but not the experimental data points themselves, are compared with each other [39].

To establish a functional relationship between the mechanical properties and relative density of porous structures, the power law has been used. In the case of the compressive properties in relation to the structure relative density, as presented in Figure 7, the exponent of the power law fitted to the experimental data points was found to vary between 0.93 and 2.34 for the elastic gradient, between 1.28 and 2.15 for the first maximum stress, between 1.75 and 3.5 for the plateau stress, between 1.21 and 2.31 for the yield stress, and between 2.18 and 73 for energy absorption (Figure 7). In other words, the exponent of the power law could be used to generalize the relationship between the structure relative density and the compressive properties of the chosen porous structures with different types of unit cells.

Complexity in quantifying the relationships between the mechanical properties and geometric parameters of scaffolds arises from the anisotropic mechanical behaviors of most unit-cell based porous structures. Weißmann et al. [115] studied the effects of the anisotropy of unit cell array orientation on the mechanical properties of scaffolds. It is worth noting that the authors presented a formula linking the Young’s modulus of the matrix material with the Young’s modulus and porosity of the scaffold. This relationship was further confirmed by the results of Wieding et al. [35] and Yavari et al. [116]. In order to define AM process parameters accurately and appropriately, the geometric design space of scaffolds for mechanical research was proposed, based on the imposed constraints of manufacturing, pore size and porosity. The experimental results indicated that the manufacturing inaccuracy led to reductions in porosity and pore size and octet truss samples with high porosity and small cell sizes were sensitive to manufacturing irregularities.

In addition to the Young’s modulus and strengths, the energy absorption capacity of lattice structure is another important performance index. Campanelli et al. compared the energy absorption capacities of the lattice structures with variable cells, truss sizes and vertical bars as reinforcements [117]. The maximum load-bearing capacity and maximum energy absorbed per unit mass were found to be achievable by adjusting the unit cell parameters.

In addition to static mechanical properties, the fatigue behavior of SLM scaffolds is considered of particular importance, because most of BTE scaffolds are subjected to cyclic loading [38]. As compared to other types of unit cells such as truncated cuboctahedron, the cubic unit cell was found to exhibit a better fatigue resistance, while the diamond unit cell had a shortest fatigue life. Both unit cell type and porosity affected the fatigue properties. Other AM technologies such as direct metal deposition were also used in the studies on fatigue behavior. Ti-6Al-4V scaffolds manufactured by EBM exhibited even better fracture strength and crack propagation behavior than cast or wrought Ti-6Al-4V, according to the results of Seifi et al. [118].

Table 3 lists some selected geometric parameters and mechanical properties of metallic scaffolds. From the table, it can be seen that the nominal sizes of pores and struts differ significantly from the measured values and these differences do not show an obvious regularity with the type of unit cells. All the studies focused on mimicking the geometric and mechanical characteristics of trabecular bone, i.e., a Young’s modulus value of 0.2–2 GPa and a yield strength value of 2–80 MPa [121]. The experimental results indicated that metallic scaffolds with stiffness and strength values comparable to those of human bone were achievable with a proper combination of structure design and AM process parameters; a large porosity (>50%) helps lower the stiffness of the matrix material and provide sufficient space for tissue ingrowth, resulting good and permanent fixation of the implant in the surrounding bone tissue. However, these studies have been confined to in vitro mechanical testing of square/cylinder specimens without functionally gradient structures.

In recent ten years, with the intensive development of AM, remarkable research has been performed to illustrate the sophisticated mechanisms of the influences of scaffold architecture and AM process parameters on the mechanical properties. However, many shortcomings are yet to overcome.

(i) In most of the studies conducted so far, uniaxial compression or tension tests have been performed and static tensile/compressive properties such as Young’s modulus, yield strength and ultimate compressive/tensile strength have been determined. However, the inner architecture and outer shape of scaffolds vary dramatically when these scaffolds are made to be used as patient-specific implants. It means that the scaffolds for clinic use have far more complex architectures and mechanical behavior. Moreover, the scaffolds for clinical applications ideally possess graded functional characteristics. In the future, mechanical testing of functionally gradient scaffolds, considering the musculoskeletal loading condition, should be performed.
(ii) Major studies on AM scaffolds have focused on the in vitro mechanical properties. More research that covers the whole line of scaffold development, from structure design to AM, in vitro mechanical tests and in vivo tests should be carried out in the future. As previously stated, the mechanical and biological properties of scaffolds are strongly related to materials, pore sizes, porosity, pore morphology, pore interconnection and unit cell type. Any variation of one of these parameters will bring about a non-negligible influence on the clinical therapeutic efficacy. Some researchers even showed a contrary tendency between in vitro and in vivo test results, as to the relationship between pore size and bone tissue regeneration [122]. Therefore, inclusion of in vivo tests is necessary.
(iii) During the AM process, a large number of metal powder particles are only partially re-melted, leading to rough surfaces. Although an irregular morphology is favorable for cell attachment, the shape irregularities inevitably make the porosity of scaffolds more uncontrollable. The application of scaffold surface treatments might notably change their mechanical properties [123]. Previous research also showed that SLM scaffolds had large thermal stresses [124]. Post heat treatment is strongly needed to eliminate residual thermal stresses [125].

## 6. In Vivo/In Vitro Studies on AM Scaffolds

Once a scaffold is fabricated using one of the AM processes, it may be directly implanted as a graft at the defect site of the body to play its biological and mechanical functions. A lot of research has been performed with a focus on the evaluation of the biological behavior of implanted scaffolds in in vivo or in vitro situations. It has been commonly acknowledged that the morphological properties of scaffolds and the addition of growth factors significantly affect cellular activities. Bone remodeling has been recognized as a process based on mechanotransduction, i.e., the ability of the bone tissue to sense mechanical loading and adapt accordingly. Generally, non-stochastic porous structures possess a superior ability for cell ingrowth to stochastic porous structures, due to a sensible strain distribution within the architecture and adequate nutrition supply of the former.

Caparros et al. [126] and St Pierre et al. [127] performed in vitro experiments using pre-osteoblastic cells to determine the relationship between the pore size of titanium scaffolds and cell adhesion, proliferation and differentiation. Scaffolds with wide pores were found to facilitate internal cell colonization and stimulate osteoblast differentiation. Human embryonic stem cell-derived mesodermal progenitors (hES-MPs) were used in another study to validate the ability of AM scaffolds to support hES-MP cell attachment and growth, but not to alter the expression of genes involved in osteogenic differentiation or affect the alkaline phosphatase activity [128].

Geometric parameters are intimately linked with cell attachment and growth [129,130,131]. Studies have been performed, based on the scaffolds with different geometric features that are manufacturable, using biocompatible materials. Cells showed different growth patterns on various structures [132,133]. Hosseini et al. [134] showed that the curvature of a geometric shape could significantly improve cell adhesion and a relatively small curvature led to a rise in bone cell growth. Their results further illustrated the importance of investigating the functional mechanisms between the geometric features of the scaffold and bone tissue growth.

Van der Stock et al. [72] incorporated bioactive gels loaded with bone morphogenetic protein-2 (BMP-2, 3 mg), fibroblast growth factor-2 (FGF-2, 0.6 mg), BMP-2, or FGF-2 (BMP-2/FGF-2, ratio 5:1) into AM scaffolds. Their study using animal models showed that the incorporation of nanostructured colloidal gelatin gels capable of time- and dose-controlled delivery of BMP-2 and FGF-2 into AM titanium scaffolds was a promising strategy to enhance and continue bone regeneration of large bone defects.

A 3D transient model of cellular growth based on the Navier–Stokes equations that simulated the body fluid flow and the stimulation of bone precursor cellular growth, attachment and proliferation as a function of local flow shear stress were presented by Zhang et al. [135]. The model’s effectiveness was demonstrated for two AM titanium scaffold architectures. The results demonstrated that there was a complex interaction between flow rate and strut architecture, resulting in partially randomized structures having a preferential impact on stimulating cell migration in 3D porous structures for higher flow rates.

## 7. Priority Areas of Further Research

In the forthcoming years, AM for BTE scaffolds will continue to develop rapidly. The combination of the biomaterials technology and AM technologies will bring about a paradigm shift to the biomedical industry and medical treatments. However, some major challenges still remain in the mechanical and biological properties of biomaterials, the design and analysis of scaffold structures and the optimization of AM process parameters.

Currently, the geometric feature size of AM scaffolds is still too large, compared to the architecture level of human bone, due to the limitations in powder particle size. The mismatch presents an obstacle to realizing the full function of the damaged bone tissue. New-generation metal powders should possess the qualities of controlled particle shape, sizes and size distribution, high mechanical performance, fast and consistent flowability and cost competitiveness. Ideal scaffolds should also eventually be biodegraded and only leave healthy bone tissue inside the human body. Therefore, biodegradable materials will be one of the focuses of the studies in the future.

The current AM processes inevitably induce defects such as shape irregularity and micro-voids into AM parts, which significantly deteriorate the overall mechanical performance of AM scaffolds. In addition, a mismatch exists between the CAD design and the final part fabricated by the AM method. Often, there are distortions in the as-built parts fabricated by SLM. In recent studies, attention has been drawn to functionally gradient scaffolds with a minimal surface and thus higher requirements are imposed on the size and morphology control of the heat-affected zone and scanning strategy optimization. Studies on AM process parameter control and processing technology improvement are in urgent need.

A variety of unit cell structures have been presented in the literature and the methodology of structural design has been improved. However, only the mechanical properties of several types of unit cells have been systematically evaluated and a unified optimization strategy and selection criteria for unit cells are still missing. Future work should be directed toward gaining a general understanding of the mechanical and biological behavior of scaffolds as affected by unit cell type and geometrical parameters. Moreover, the mechanical properties and biological properties of AM scaffolds are of equal importance and should be comprehensively considered in BTE.

The importance of porosity diversity and structural gradients within BTE scaffolds has been increasingly realized in recent years [136]. The mechanical properties of scaffolds with various porosities [102] and even some functionally graded implants have been studied [137,138]. However, a universal methodology for the design of functionally graded BTE scaffolds and automated optimization procedures have not yet been developed. With the maturing of the metal AM technology and the FE simulation technology to assist in optimizing the design of implants and functionally graded scaffolds, future research will be focused on the integration of implant and scaffold features into patient-specific devices with either abrupt transition or gradual transition in pore geometry and porosity between the solid zone and the porous zone for individual mechanical and biological functions. In this way, the full potential of AM will be exploited.

## Figures and Tables

**Table 1:** Additive manufacturing (AM) technologies, their features and applications.

Method	Process Characteristics	Applicable Metallic Materials for Bone Tissue Engineering	Advantages (+) and Disadvantages (−)	Category	Manufacturer
Powder bed and inkjet 3D printing (3DP) [45,46]	Depositing binder on metal powder Curing the binder to hold the powder together Sintering or consolidating the bound powder Infiltrating with a second metal (optionally)	Stainless steel, iron, cobalt-chromium alloy, zirconium, tungsten, etc.	Ability to create shapes that are difficult or impossible for traditional methods (+) No need for potentially extensive laser optimization experimentation (+) No heat source is used during the processing (+) No need for a build plate (+) Need post-processing (−) Considerable porosity exists (−) Not available for part reparation (−)	Binder jetting	ExOne, 3D System
Selective laser sintering (SLS) [47]	Preparing the powder bed Layer by layer addition of powder Sintering each layer according to the CAD file, using laser source	Stainless steel, cobalt-chromium alloy, titanium, etc.	No need for support (+) No post-processing is needed (+) Need heat treatment and material infiltration (−) Porous part and rough surface (−) Thermal distortion (−) Not available for part reparation (−)	Powder bed fusion	EOS
Selective laser melting (SLM) [48,49]	Thin layers (20–100 μm) of atomized fine metal powder are evenly distributed using a coating mechanism onto a substrate plate, usually metal Each 2D slice of the part geometry is fused by selectively melting the powder The process is repeated layer after layer until the part is complete	Stainless steel, iron based alloys, titanium, gold, silver, etc.	Capable of fully melting the powder material, producing fully dense near net-shape components without the need for post-processing (+) High processing precision (≤10 μm) (+) Support needed where necessary (−) High quality demands for metal powders and limited part size (−) Distortion caused by residual thermal stress (−) Not available for part reparation (−)	Powder bed fusion	SLM Solutions
Electron beam melting (EBM) [50,51]	The EBM machine reads data from a 3D CAD model and lays down successive layers of powder These layers are melted, utilizing a computer controlled electron beam under vacuum	Titanium alloys, cobalt chromium alloy	Kinetic energy transfer and preheating the powder result in lower thermal stresses (+) Vacuum environment; metal does not oxidize easily (+) No support needed (+) Complex internal cavities not possible due to preheating/sintering process (−) Rougher texture and less precise than laser beam manufacturing (−)	Powder bed fusion	Arcam
Direct metal laser sintering (DMLS) [52]	Spreading a very thin layer of metal powder across the surface that is to be printed Laser slowly and steadily moves across the surface to sinter powder Additional layers of powder are then applied and sintered	Stainless steel, titanium, etc.	Parts free from residual stresses and internal defects (+) Expensive; limited its use to high-end applications (−) Not suitable for low ductility materials (−) Heating stage needed for low ductility materials (−)	Powder bed fusion	Stratasys
Direct metal deposition (DMD) [45,53,54]	Powder is melted using laser or other kind of energy at the nozzle and then deposited layer by layer	Iron, titanium, etc.	Part size is not limited to bed size; large metal parts (+) No limitation in processing space (+) Available for part reparation (+) Poor surface finish (−)	Direct energy deposition	Optomec, TWI
Electron beam additive manufacturing (EBAM) [45,53]	Convert CAD model to CNC code Electron beam gun deposits metal, via a powder or wire feedstock, layer by layer, until the part reaches the near-net shape Finish heat treatment and machining	Titanium, stainless steels, zinc alloy, tantalum, tungsten, etc.	Part size is not limited to bed size; large metal parts (+) Good material utilization (+) Multiple wire feed nozzles can be utilized with a single EB gun (+) Lower processing accuracy than powder bed AM and poor surface finish (−)	Direct energy deposition	Sciaky, Efesto

**Table 2:** Qualitative comparison between different AM processes.

AM Process	Resolution	Build Speed	Surface Roughness	Power Efficiency	Build Volume	Residual Stress	Cost
3DP	Poor	Fast	Poor	-	Big	Low	Low
SLS	Good	Slow	Excellent	Poor	Small	High	High
SLM	Good	Slow	Excellent	Poor	Small	High	High
EBM	Moderate	Fast	Good	Good	Small	Moderate	High
DMLS	Good	Slow	Excellent	Poor	Small	Low	High
DMD	Poor	Fast	Poor	Poor	Big	High	Moderate
EBAM	Moderate	Moderate	Good	Good	Small	Moderate	High

**Table 3:** Mechanical properties of scaffolds made of different types of unit cells.

Unit Cell	Material	Pore Size (μm)		Strut Diameter (μm)		Porosity (%)		Young’s Modulus (GPa)	Yield Stress (MPa)	References
		Nominal	Measured	Nominal	Measured	Nominal	Measured			
Cube	Ti-6Al-4V	348~720	451~823	1452~1080	1413~1020	65~90	63~87	1.76~4.62	29~110	[39,98]
	Ti-6Al-4V	550, 800	-	300, 400	-	70.3~70.7	70.2~68.7	5.10~6.70	155~164 (UCS)	[35]
	Ti-6Al-4V	1000~2040	765~1020	450, 800	466~941	60.91~75.83	49.75~59.32	0.57~2.92	7.28~163.02	[119]
Diamond	Ti-6Al-4V	277~600	240~564	923~600	958~641	89~63	89~64	0.39~3.30	7~70	[39]
	Ti-6Al-4V	-	670~1820	-	420~540	-	87~60	0.4~6.5	11.4~99.7	[120]
Truncated cube	Ti-6Al-4V	1720~1370	1625~1426	180~530	331~620	94~76	91~80	0.99~3.19	10~40	[39]
Truncated cuboctahedron	Ti-6Al-4V	876~807	862~1049	324~564	862~1049	82~64	81~64	2.37~4.62	25~100	[39]
Rhombic dodecahedron	Ti-6Al-4V	1250~950	1299~1058	250~550	246~506	90~66	89~68	0.22~2.97	7~88	[39]
	Ti-6Al-4V	-	-	-	67~129	-	84~67	0.55	-	[37]
Rhombicuboctahedron	Ti-6Al-4V	820~670	877~794	380~530	348~438	84~64	89~68	2.23~4.40	39~93	[39]
Dodecahedron	Ti-6Al-4V	~	150	~	500	-	80	1.22	12.7	[32]
	CP-Ti	450, 500	~	120, 170, 230	-	-	66~82	0.58~2.61	8.6~36.5	[26]
	Ti6-Al-4V	500, 450	560, 486	120, 170	140, 216	-	68~84	0.55~3.49	15.8~91.8	[115]
Tetrahedron	Ti-6Al-4V	500	-	0.2~0.39	-	50~75	-	4.3~1.9	57~156	[30]
Octet truss	Ti-6Al-4V	770	-	0.2~0.4	-	50~75	-	4.6~1.2	34~172	[30]
Twist struts	Ti-6Al-4V	-	-	0.90, 1.10	-	55~60	55~61	3.4~26.3	103~402	[115]
Gyriod TPMS	Ti-6Al-4V	-	560~1600	-	-	-	80~95	0.13~1.25	6.50~81.30	[27]
Diamond TPMS	Ti-6Al-4V	-	480~1450	-	-	-	80~95	0.12~1.25	4.66~69.21	[27]
